# LW6, a hypoxia-inducible factor 1 inhibitor, selectively induces apoptosis in hypoxic cells through depolarization of mitochondria in A549 human lung cancer cells

**DOI:** 10.3892/mmr.2015.3862

**Published:** 2015-05-27

**Authors:** MARIKO SATO, KATSUMI HIROSE, IKUO KASHIWAKURA, MASAHIKO AOKI, HIDEO KAWAGUCHI, YOSHIOMI HATAYAMA, HIROYOSHI AKIMOTO, YUICHIRO NARITA, YOSHIHIRO TAKAI

**Affiliations:** 1 Department of Radiology and Radiation Oncology, Hirosaki University Graduate School of Medicine, Hirosaki, Aomori 036-8562, Japan; 2 Department of Radiological Life Sciences, Hirosaki University Graduate School of Health Sciences, Hirosaki, Aomori 036-8564, Japan

**Keywords:** hypoxia-inducible factor 1 inhibitor, hypoxia, apoptosis, A549

## Abstract

Hypoxia-inducible factor 1 (HIF-1) activates the transcription of genes that act upon the adaptation of cancer cells to hypoxia. LW6, an HIF-1 inhibitor, was hypothesized to improve resistance to cancer therapy in hypoxic tumors by inhibiting the accumulation of HIF-1α. A clear anti-tumor effect under low oxygen conditions would indicate that LW6 may be an improved treatment strategy for cancer in hypoxia. In the present study, the HIF-1 inhibition potential of LW6 on the growth and apoptosis of A549 lung cancer cells in association with oxygen availability was evaluated. LW6 was observed to inhibit the expression of HIF-1α induced by hypoxia in A549 cells at 20 mM, independently of the von Hippel-Lindau protein. In addition, at this concentration, LW6 induced hypoxia-selective apoptosis together with a reduction in the mitochondrial membrane potential. The intracellular reactive oxygen species levels increased in LW6-treated hypoxic A549 cells and LW6 induced a hypoxia-selective increase of mitochondrial O2^•−^. In conclusion, LW6 inhibited the growth of hypoxic A549 cells by affecting the mitochondria. The inhibition of the mitochondrial respiratory chain is suggested as a potentially effective strategy to target apoptosis in cancer cells.

## Introduction

According to Cancer Statistics In Japan 2012, it was estimated that ~357,000 people died from cancer in 2011 in Japan (1). The treatment strategies for cancer involve surgical treatment, chemotherapy/molecular targeted therapy and radiotherapy; however, cancer cells acquire resistance when treatment is prolonged and hypoxic conditions serve a role in this acquired resistance (2). Furthermore, hypoxia has been previously reported to be involved in metastasis and recurrence of cancer (3–10). Therefore, hypoxia has become a key target of cancer therapy.

The transcription factor hypoxia-inducible factor-1α (HIF-1α) is accumulated in tumor cells under hypoxic conditions and is involved in the acquired resistance towards cancer therapy and adaptation to hypoxia (2). HIF-1α moves into the nucleus and promotes the expression of numerous genes involved in angiogenesis, cell proliferation, glucose metabolism and apoptosis (2). Furthermore, the intracellular accumulation of HIF-1α inhibits the production of reactive oxygen species (ROS) induced by hypoxic stress, and HIF-1α serves an important part in the adaptation of cells to hypoxia (11,12). In the presence of oxygen, HIF-1α is degraded by the ubiquitin-proteasome system subsequent to hydroxylation by the von Hippel-Lindau (VHL) protein. Conversely, non-hydroxylated HIF-1α enters the nucleus to form a heterodimer with the constitutively expressed HIF-1β (13). The HIF-1 heterodimer binds to the hypoxia-response element (HRE), thereby activating the expression of numerous hypoxia-response genes, including the pro-angiogenic growth factor vascular endothelial growth factor (VEGF) (13).

Various types of small molecule inhibitors of HIF-1 have been developed and studied previously (14–16). One example, LW6, has been reported to upregulate the VHL protein (17). As a result, the transcriptional activity of the hypoxia-response genes is downregulated due to the marked degradation of HIF-1α. LW6 is therefore hypothesized to improve resistance to cancer therapy in hypoxia. Previous studies have demonstrated that LW6 exerts marked anti-tumor efficacy *in vivo* and causes reductions in HIF-1α expression levels in mice carrying xeno-grafts of HCT116 cells (17). However, it is not clear whether the difference of anti-tumor efficacy is associated with the oxygen levels. The aim of the present study was to investigate whether LW6 enhances cytotoxicity selectively in hypoxic cells through depolarization of the mitochondrial membrane potential (MMP). These results suggested that agents which are able to depolarize the MMP, such as LW6, may represent a novel therapeutic strategy to be used on hypoxic cells that survive other cancer therapies.

## Materials and methods

### Materials

Dulbecco's modified Eagle's medium (DMEM) was obtained from Sigma-Aldrich (St. Louis, MO, USA). Penicillin and streptomycin were obtained from Gibco-BRL (Invitrogen Life Technologies, Carlsbad, CA, USA) and fetal bovine serum (FBS) was obtained from GE Healthcare (Little Chalfont, UK). LW6 was purchased from Merck Millipore (Darmstadt, Germany) and diluted in dimethyl sulfoxide (DMSO; Wako Pure Chemical Industries, Ltd., Osaka, Japan). Mouse monoclonal anti-HIF-1α antibody (ab1) was obtained from Abcam (Cambridge, UK) and goat polyclonal anti-actin antibody (sc-1615) was obtained from Santa Cruz Biotechnology (Dallas, TX, USA).

### Cell culture and growth conditions

The human lung adeno-carcinoma cell line A549 was grown in DMEM supplemented with penicillin, streptomycin and 10% heat-inactivated FBS at 37°C in a humidified atmosphere containing 5% CO_2_. Hypoxia was defined as 1% oxygen, which was achieved by culturing cells in modular incubator chambers (Billups-Rothenberg, Inc., Del Mar, CA, USA), which were flushed with gas mixtures (95% nitrogen/5% carbon dioxide) and sealed to maintain hypoxia.

Cells were seeded into 35-mm dishes (Iwaki, Chiba, Japan) at 2×10^5^ cells/dish with 1.5 ml medium containing LW6 for 12 h. Cells were incubated under normoxia or hypoxia for 36 h and were then assessed for the expression of HIF-1α and the ratio of apoptotic cells. To analyze active caspase-3, the cells treated with LW6 for 12 h were exposed to hypoxia or normoxia for 48 h and the cells were then analyzed.

### Cell viability analysis

Cells were incubated in 96-well ELISA Plates (Iwaki) with 100 *μ*l culture medium at 2×10^5^ cells/ml with or without LW6 for 24 h. Cell viability was assessed by the dimethyl thiazolcarboxy-methoxyphenylsulfophenyltetrazolium (MTS) assay performed using a CellTiter 96^®^ AQueous One Solution Cell Proliferation Assay kit (Promega Corporation, Madison, WI, USA) according to the manufacturer's instructions. The absorbance was measured at 490 and 620 nm using a microplate reader (iMark™; Bio-Rad Laboratories, Inc., Hercules, CA, USA). For the trypan blue dye. exclusion test, cells were stained by phosphate-buffered saline (PBS) containing 0.1% trypan blue (Nacalai Tesque, Inc., Kyoto, Japan). Cell viability was assessed by counting the number of unstained cells using the TC20™ automated cell counter (Bio-Rad Laboratories, Inc.).

### Western blot analysis

Cells were lysed using Cell Lysis Buffer (Cell Signaling Technology, Inc., Danvers, MA, USA) and phenylmethanesulfonylfluoride (Sigma-Aldrich). Cell lysates and pre-stained molecular weight markers were separated by SDS-PAGE with 12% Mini-PROTEAN^®^ TGX™ precast gels (Bio-Rad Laboratories, Inc.), followed by transfer onto polyvinylidene fluoride membranes with Trans-Blot^®^ Turbo™ (Bio-Rad Laboratories, Inc.). The membranes were blocked in Tris-buffered saline with 0.1% Tween-20 containing 5% milk and then incubated with various primary antibodies diluted in blocking buffer. Mouse monoclonal anti-HIF-1a antibody (cat. no. 610959; BD Biosciences) at a dilution of 1:200, rabbit monoclonal anti-histone H1 antibody (cat. no. ab125027; Abcam) at a dilution of 1:200, goat polyclonal anti-actin antibody (cat. no. sc-1615; Santa Cruz Biotechnology) at a dilution of 1:1,000, rabbit monoclonal anti-VEGF antibody (cat. no. ab52917; Abcam) at a dilution of 1:200, rabbit polyclonal anti-VHL antibody (cat. no. sc-5575; Santa Cruz Biotechnology) at a dilution of 1:200 were incubated for 1 h. The blots were then washed with Tris-buffered saline containing 0.1% Tween-20 (Bio-Rad Laboratories, Inc.) three times and incubated with horseradish peroxidase-conjugated donkey anti-mouse IgG (cat. no. sc-2314; Santa Cruz Biotechnology) at a dilution of 1:5,000, donkey anti-rabbit IgG (cat. no. sc-2313; Santa Cruz Biotechnology) at a dilution of 1:5,000, or donkey anti-goat IgG (cat. no. sc-2056; Santa Cruz Biotechnology) at a dilution of 1:5,000 in blocking buffer for 1 h. Membranes were washed three times and immunoreactivity was visualized using a chemiluminescence Molecular Imager^®^ ChemiDoc™ XRS+ system (Bio-Rad Laboratories, Inc.) according to the manufacturer's instructions.

### Detection of HIF-1α

HIF-1α was detected using the fluorescein isothiocyanate (FITC)-conjugated monoclonal active HIF-1α antibody (ab1; Abcam) according to the manufacturer's instructions. Briefly, cells were collected with 0.1% trypsin under hypoxia. Following incubation on ice for 20 min, the cells were washed with ice-cold PBS and suspended in Cytofix/Cytoperm™ solution (BD Biosciences). Subsequently, following incubation on ice for 20 min, the cells were centrifuged for 3 min at 200 × g, pelletted and the supernatant was aspirated and then washed with wash buffer at room temperature. The cells were suspended in the wash buffer containing the anti HIF-1α antibody at a dilution of 1:200 for 30 min on ice in the dark. The cells were washed and incubated with secondary donkey anti-mouse immuno-globulin G-FITC (sc-2099; Santa Cruz Biotechnology, Inc.) antibody at a dilution of 1:200 for 30 min on ice in the dark, and were then analyzed using a Cell Lab Quanta™ SC flow cytometer (Beckman Coulter, Inc., Fullerton, CA, USA). The results were analyzed using FlowJo software, version 7.6.5 (TreeStar, Inc., Ashland, OR, USA).

### Detection of active caspase-3

The detection of active caspase-3 was performed using the FITC-conjugated monoclonal active caspase-3 antibody apoptosis kit I (BD Biosciences, Franklin Lakes, NJ, USA) according to the manufacturer's instructions. Briefly, cells were washed with ice-cold PBS and suspended in Cytofix/Cytoperm™ solution. Subsequent to incubation on ice for 20 min, the cells were pelleted, aspirated and then washed with wash buffer at room temperature. The cells were suspended in the wash buffer containing 5% (v/v) FITC-conjugated anti-active caspase-3 antibody for 40 min at room temperature in the dark. The cells were then washed with the wash buffer and analyzed using a Cell Lab Quanta™ SC flow cytometer.

### Annexin V staining

The detection of apoptotic cells was performed using the TACS Annexin V-FITC Apoptosis Detection kit (Trevigen, Inc., Helgerman Ct., MD, USA) according to the manufacturer's instructions. Briefly, following a 12-h incubation in a 35-mm culture dish (Iwaki) under hypoxia or normoxia, the cells were harvested, washed and suspended with 100 *μ*l binding buffer, and were then stained with Annexin V-FITC for 10 min on ice in the dark. Following washing with the binding buffer, the cells were re-suspended in the buffer with propidium iodide (PI). Apoptotic cells were determined using a Cell Lab Quanta™ SC flow cytometer. Subsequent to excluding the PI-positive cells from gating, the fraction of Annexin V-positive cells was evaluated.

### Cell cycle analysis

The cell cycle phase distribution was analyzed using PI staining according to the manufacturer's instructions. Briefly, following a 12-h incubation in a 35-mm culture dish (Iwaki) under hypoxia or normoxia, the cells were harvested. Subsequent to washing with the binding buffer, the cells were re-suspended in the buffer with PI. Stained cells were analyzed for PI fluorescence using a Cell Lab Quanta™ SC flow cytometer.

### Determination of mitochondrial membrane potential (MMP)

The MMP was determined by staining with JC-1 (Setareh Biotech, Eugene, OR, USA) according to the manufacturer's instructions. Briefly, cells were washed in PBS and incubated with 4 *μ*M JC-1 at 37°C for 10 min. The cells were then washed in PBS and were observed using a BZ-9000 fluorescence microscope (Keyence, Osaka, Japan). Cells were then harvested and stained with JC-1 as described above. The cells were then re-suspended in PBS and analyzed using a Cell Lab Quanta™ SC flow cytometer.

### Measurement of mitochondrial O_2_^•−^

The MitoSOX™ RED Mitochondrial O_2_^•−^ Indicator (Invitrogen Life Technologies) was used to detect mitochondrial O_2_^•−^. Briefly, the cells were incubated with 20 *μ*M LW6 under normoxia or hypoxia, were washed in PBS and were then incubated with 5 *μ*M MitoSOX™ RED at 37°C for 30 min according to the manufacturer's instructions. The cells were then washed, re-suspended in 300 *μ*l PBS and analyzed using a Cell Lab Quanta™ SC flow cytometer.

### Statistical analysis

The significance of the differences was determined using Student's two-tailed t-test and Welch's t-test depending on the data distribution. P<0.05 was considered to indicate a statistically significant difference. Excel 2007 software (Microsoft Corporation, Redmond, WA, USA) with the add-in software Statcel 3 was used for statistical analysis.

## Results

### LW6 inhibits HIF-1α expression induced by hypoxia

**Figure 1:**
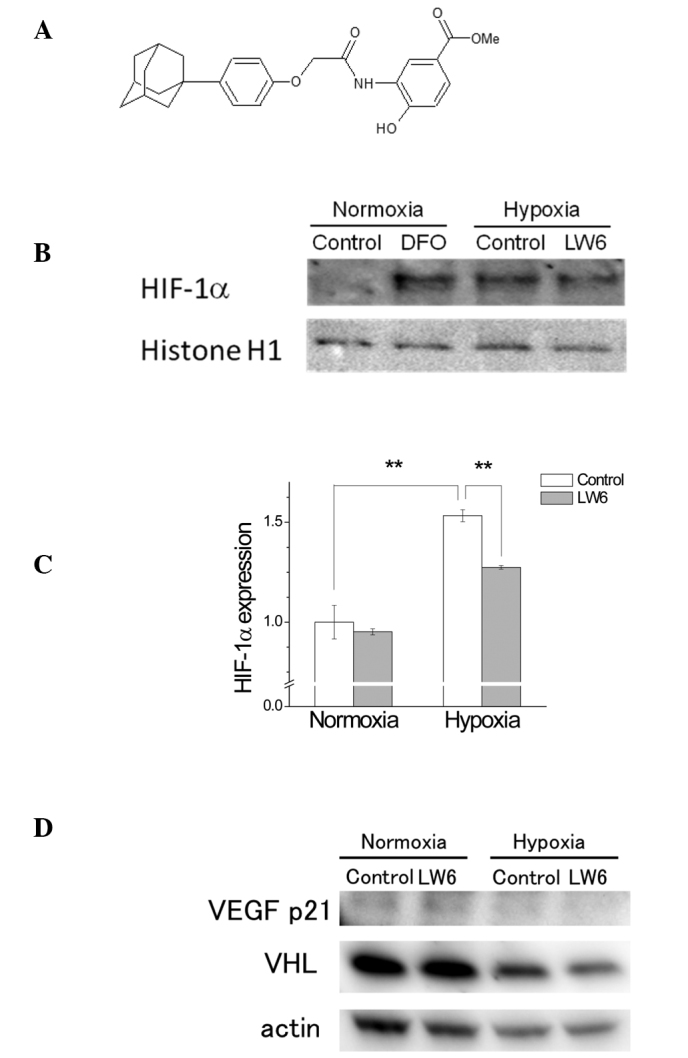
LW6 inhibits hypoxic induction of HIF-1α expression. (A) Chemical structure of LW6, 3-[2-(4-adamantan-1-yl-phenoxy)-acetylamino]-4-hy-droxy-benzoic acid methyl ester. The effects of LW6 on HIF-1α expression levels were determined by (B) western blot analysis and (C) flow cytometry. Cells were pre-treated with 20 *μ*M LW6 for 12 h and incubated under nor-moxic or hypoxic conditions for (B) 8 h or (C) 20 h. DFO, a hypoxia-mimetic agent, was observed to be able to increase HIF-1α expression levels. (D) Expression of VEGF and VHL was assessed using western blot analysis. Cells were pretreated with 20 mM LW6 for 12 h and incubated for 8 h. Values are expressed as the ratio of treated vs. untreated cells. The results are presented as the mean ± standard error of three different experiments. ^**^P<0.01 vs. untreated cells, determined by Student's t-test. HIF-1α, hypoxia-inducible factor 1α; DFO, desferrioxamine.

Although LW6 is synthesized as an (aryloxyacetylamino)benzoic acid derivative (Fig. 1A) and has the potential to inhibit the expression of HIF-1α in the HCT116 human colon cancer cell line (17), its mechanism of action has remained to be fully elucidated. A preliminarily investigation of the cytotoxicity of different concentrations of LW6 against A549 cells was undertaken. A549 cells were incubated with 5–100 *μ*M LW6 for 24 h and the cytotoxic concentration of LW6 was determined by MTS assay. It was observed that 100 *μ*M LW6 significantly reduced the cell viability (0.73±0.02; P<0.01). No cytotoxic effects were observed with concentrations of up to 50 mM in A549 cells (data not shown).

The increase of HIF-1α expression under hypoxia was reported to be inhibited by LW6 through the overexpression of VHL, leading to the inhibition of tumor angiogenesis in the HCT116 human colon cancer cell line (17). The potential inhibitory effect of LW6 on the expression of HIF-1α was investigated in A549 cells. Although the cells incubated under hypoxia for 36 h exhibited increased expression levels of HIF-1α, the treatment with LW6 partially reversed this hypoxia-induced HIF-1α expression (Fig. 1B and C). LW6 had no effect on the expression of HIF-1α in the normoxic group. The expression levels of VHL and VEGF were not attenuated by LW6 treatment in either hypoxia or normoxia (Fig. 1D). These results suggested that LW6 had an inhibitory effect on HIF-1α expression independent of the upregulation of VHL.

### LW6 promotes apoptosis preferentially in hypoxic cells

**Figure 2:**
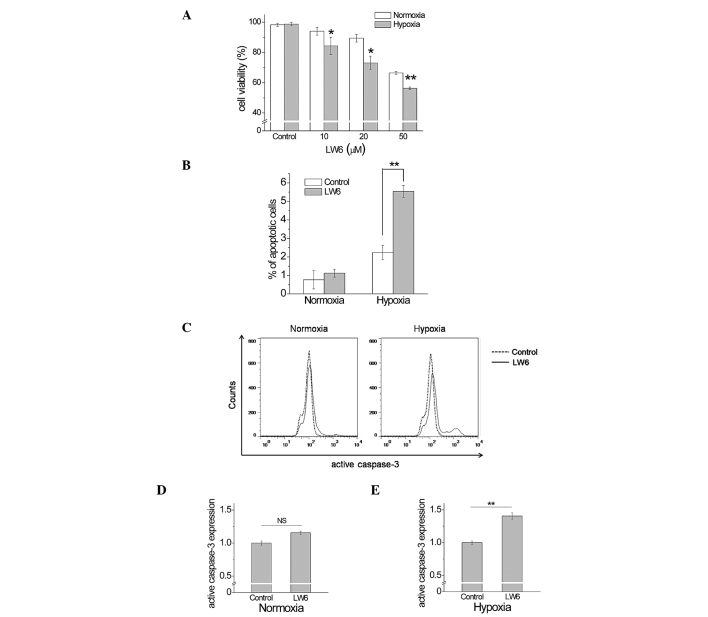
LW6 promotes apoptosis in hypoxic A549 cells. (A) The cytotoxicity of LW6 toward A549 cells under hypoxia and normoxia. A significant difference between normoxic and hypoxic cells was observed. (B) Effects of LW6 on the level of apoptosis. Cells were pre-treated with 20 *μ*M LW6 for 12 h and incubated under normoxic or hypoxic conditions for 36 h. (C-E) Effects of LW6 on active caspase-3 expression levels. Cells were pre-treated with 20 *μ*M LW6 for 12 h and incubated under normoxic or hypoxic conditions for 48 h. Cells were then analyzed by flow cytometry. (D and E) Quantified results as the ratio of treated vs. untreated cells. Values are expressed as the mean ± standard error of three different experiments. ^*^P<0.05 and ^**^P<0.01 vs. control cells, determined by Student's t-test.

**Figure 3:**
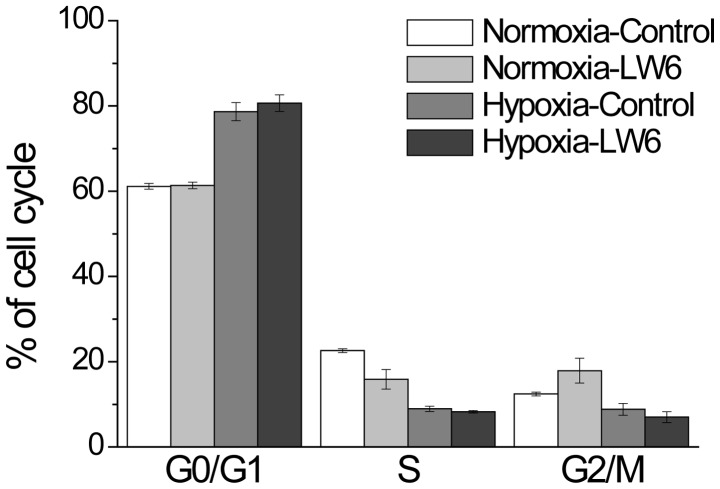
LW6 was demonstrated to have no effect on the cell cycle. Cells were pre-treated with 20 *μ*M LW6 for 12 h and incubated under normoxic or hypoxic conditions for 36 h. Cells were subsequently analyzed by flow cytometry. Values are expressed as the mean ± standard error.

Next, the present study investigated whether the cell death induced by LW6 is dependent on the oxygen levels that may be modulated by mitochondrial respiration. Although the exposure of cells to 20 *μ*M LW6 for 24 h resulted in no significant toxicity for the metabolic activity of the mitochondria in the preliminarily MTS assay (data not shown), cell death was induced by the 48-h exposure to LW6. The cell viability was significantly reduced in a dose-dependent manner for LW6 under hypoxia compared with normoxic conditions (Fig. 2A). The cells pre-treated with LW6 under hypoxic conditions exhibited a significant increase in the percentage of apoptotic cells compared with that of non-treated cells under hypoxic conditions (5.54±0.32% vs. 2.24±0.39%, P<0.01, Fig. 2B). No significant alterations were observed between pre-treated and non-treated cells under normoxic conditions (1.12±0.20% vs. 0.77±0.49%, P>0.05). The expression levels of active caspase-3 were investigated in order to analyze whether active caspase-3 was involved in the promotion of apoptosis by LW6. While no significant difference was observed in active caspase-3 expression between pre-treated and non-treated cells under normoxic conditions (1.00±0.031 vs. 1.15±0.024; P>0.05; Fig. 2C and D), cells pre-treated with LW6 under hypoxic conditions displayed a significant increase in active caspase-3 expression compared with that in non-treated cells (1.00±0.024 vs. 1.40±0.048; P<0.01; Fig. 2C and E). These results suggested that active caspase-3 expression participates in the promotion of apoptosis selectively induced by LW6 in hypoxic A549 cells. Although the proportion of G_1_ cells was marginally increased under hypoxic conditions compared with that under normoxic conditions, treatment with LW6 presented no additive effect on cell cycle arrest (Fig. 3).

### LW6 induces ROS formation through the depolarization of MMP in hypoxic cells

**Figure 4:**
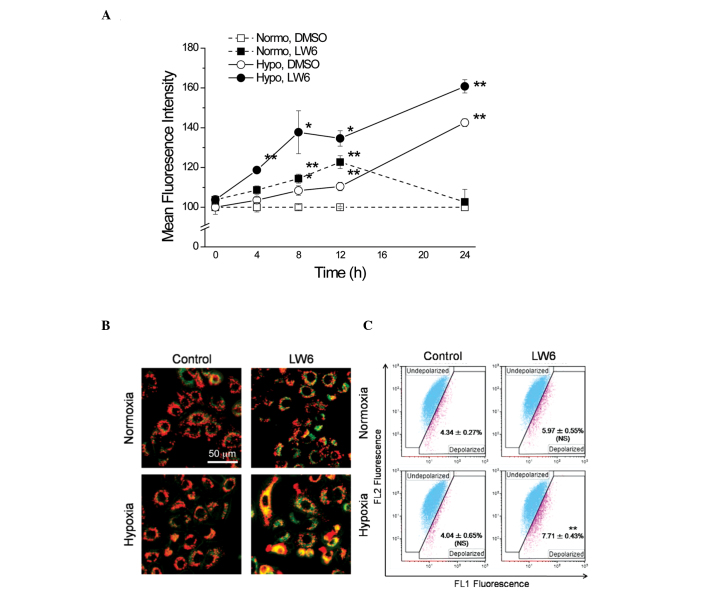
LW6 induces oxidative stress through mitochondria. (A) Alterations in the production of mitochondrial reactive oxygen species. Cells were pre-treated with 20 *μ*M LW6 for 12 h and then exposed to hypoxia or normoxia. Mitochondrial superoxide levels were detected by MitoSOX™ RED staining using flow cytometry. (B and C) Cells were pre-treated with 20 *μ*M LW6 for 12 h and then exposed to 8 h hypoxia or normoxia. The cells were then stained with 4 *μ*M JC-1. The fluorescence of JC-1 was detected by (B) microscopy and (C) flow cytometry. Values are expressed as the mean ± standard error of three different experiments. ^*^P<0.05 and ^**^P<0.01 vs. untreated normoxic cells, determined by Student's or Welch's t-test.

The activity of the mitochondrial respiratory chain has been previously reported to be inhibited by HIF-1α (18). Malate dehydrogenase 2 (MDH2), a critical enzyme involved in the aerobic metabolism of the mitochondria and the citric acid cycle, has been identified as a target protein of the HIF-1 inhibitor LW6 (19). The overproduction of intracellular ROS by mitochondria has been detected in almost all cancer cells, and the production of ROS is directly associated with the efficacy of mitochondrial oxygen utilization (20). Therefore, whether LW6 affects the production of intracellular ROS via the mitochondria was evaluated. The fluorescence intensity of MitoSOX™ RED was examined using flow cytometry and subsequently, the time-dependent alterations in the production of mitochondrial O2^•−^ were assessed. The exposure to hypoxia significantly increased mitochondrial ROS production and maintained mitochondrial ROS at high levels. Although LW6 increased mitochondrial ROS production, the combination with hypoxia induced a marked increase in ROS production and this high level was maintained up until 24 h (Fig. 4A).

Subsequently, the MMP was measured with JC-1, which also suggested that an alteration in oxygen utilization efficiency between normoxia and hypoxia results in attenuation of MMP. As presented in Fig. 4B, treatment 20 *μ*M LW6 combined with hypoxia for 8 h induced a significant reduction in MMP in A549 cells. Treatment with LW6 under normoxia was not able to significantly attenuate the MMP, thus suggesting that the effect of LW6 is dependent on hypoxic conditions (Fig. 4C).

## Discussion

In the present study, the potential of LW6 for inducing apoptosis in normoxic and hypoxic cells was examined. The results suggest that LW6 preferably induces apoptosis in hypoxic cells. LW6 was previously reported to inhibit the accumulation of HIF-1α in hypoxic cells through the upregulation of VHL protein (17). The VHL protein maintains a low level of HIF-1α expression in normoxic cells through an ubiquitin-dependent protein degradation mechanism. HIF-1α accumulates in cells under hypoxia induced by the rapid growth and increase in tumor oxygen consumption, enabling the cells to adjust to the state of hypoxia through gene expression (21). Therefore, necrotic cell death and apoptosis of cancer cells may be induced by the deletion of HIF-1α. Supporting this hypothesis, the induction of apoptosis accompanied by downregulation of HIF-1α was observed in hypoxic cells. In agreement with the results of the present study, it has been previously reported that reagents able to inhibit the expression of HIF-1α in hypoxic cells promote apoptosis (22,23). Conversely, HIF-1 is involved in hypoxia-induced apoptosis via the stabilization of p53, through the combination of HIF-1 with p53 ubiquitin ligase mdm2, or by a direct interaction between HIF-1 and p53 (24,25). An additional mechanism proposed for HIF-1-mediated apoptosis is the induction of the expression of the pro-apoptotic protein (B-cell lymphoma 2/adenovirus E1B 19-kDa interacting protein 3 (BNIP3) gene by HIF-1, through the binding of the transcription factor to the HRE sequence in the BNIP3 promoter, thereby resulting in the BNIP3 protein initiating apoptosis and inducing necrosis (26,27). However, high concentrations of HIF-1α resulting from activation of the phosphoinositide 3-kinase/Akt pathway have been reported to potentiate resistance to hypoxia-induced apoptosis in a pancreatic cancer cell line (28). Previous studies, in addition to the results of the present study, suggested that HIF-1 enables adaptation to the hypoxic conditions by maintaining the balance between pro-apoptotic and anti-apoptotic factors.

Hypoxia results in hyperpermeability of the inner mitochondrial membrane, which leads to the release of cytochrome C (29). It was suggested that inhibition of the electron transport chain at the inner mitochondrial membrane induces apoptosis (29). Lee *et al* (17) revealed that LW6 is a specific inhibitor of MDH2 (17). As MDH2 is known to serve a significant role in the citric acid cycle at the mitochondrial membrane, LW6 indirectly reduces the activity of the mitochondrial respiratory chain through the inhibition of MDH2. It was hypothesized that the effect of LW6 on MDH2 activity indirectly inhibits the electron transport chain, thus leading to apoptosis. In addition, in the present study the intracellular ROS levels in the hypoxic A549 cells treated with LW6 were significantly increased. ROS production resulting from mitochondrial dysfunction may induce the release of cytochrome C, which subsequently leads to cell death. In accordance with this, it was observed in the present study that the loss of MMP is accompanied by the production of mitochondrial O2^•−^ in hypoxic cells treated with LW6. Although the influence of LW6 on ROS production remains to be fully elucidated, the results of the present study suggested that the hypoxia-selective apoptotic effects are closely associated with the loss of MMP along with the dysfunction of mitochondria and increased ROS levels.

In conclusion, LW6 was demonstrated to be able to inhibit the accumulation of HIF-1α and induce apoptosis through depolarization of the MMP in hypoxic cells. The present study suggested that LW6 may be useful in the induction of cell death in hypoxic cells that have developed resistance to chemotherapy and radiotherapy. LW6 provides novel insight into cancer therapy strategy, particularly for the hypoxic cancer cells commonly observed in tumor tissues.
